# Identification of microplastics in wastewater after cascade filtration using Pyrolysis-GC–MS

**DOI:** 10.1016/j.mex.2019.100778

**Published:** 2019-12-19

**Authors:** Matin Funck, Aylin Yildirim, Carmen Nickel, Jürgen Schram, Torsten C. Schmidt, Jochen Tuerk

**Affiliations:** a Institut für Energie – und Umwelttechnik e.V. (IUTA, Institute of Energy and Environmental Technology), Bliersheimer Str. 58-60, 47229 Duisburg, Germany; b Instrumental Analytical Chemistry (IAC), University of Duisburg-Essen, Universitätsstrasse 5, 45141 Essen, Germany; c Instrumental Analytical and Environmental Chemistry, Niederrhein University of Applied Sciences, Frankenring 20, Krefeld, Germany; d Centre for Water and Environmental Research (ZWU), University of Duisburg-Essen, Universitätsstrasse 2, 45141 Essen, Germany; e IWW Water Centre, Moritzstraße 26, 45476 Mülheim an der Ruhr, Germany

**Keywords:** N/A, Microplastic, Microplastic sampling, Cascadic filtration, Pyrolysis, GC–MS, Microplastic filtration

## Abstract

The combination of a representative microplastic sampling method and a fast-quantitative analysis using Pyrolysis-GC–MS (Py-GC–MS) for investigation of the microplastic load and mass balances is presented in this work. A representative microplastic filtration requires a method allowing quick extraction of the sample. The developed steel based cascadic microplastic filtration uses steel basket filters with mesh sizes of 100 μm, 50 μm and 10 μm and a mean recovery of 86 % without cross contamination was achieved. Thermoanalytical methods have the advantage of minimal sample preparation with short analysis times. The presented platinum filament-based Py-GC–MS method requires little sample preparation and quantification limits for polystyrene (PS) and polyethylene (PE) were 0.03 μg and 1 μg absolute, respectively. The relative standard deviation of the analytical method is 11 %. The combined method allows representative sampling and analysis of MP from water bodies and waste water treatment plants within 48 h.

• Presentation of a validated steel based cascadic microplastic filtration plant.
• Fast and reproduceable Py-GC–MS analysis method for microplastic.
• Py-GC–MS allows microplastic analysis with little sample preparation.

## Graphical abstract

**Figure:**
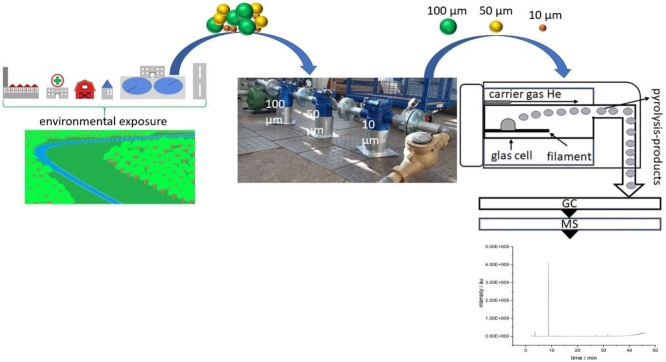

**Specification Table**

**Table:** 

Subject Area:	Environmental Science
More specific subject area:	Microplastic sampling and analysis
Method name:	N/A
Name and reference of original method:	N/A.
Resource availability:	Pyrola 2000 (Pyrolab; Lund, Sweden)

## Method details

### Microplastic filtration plant

**Fig. 1:**
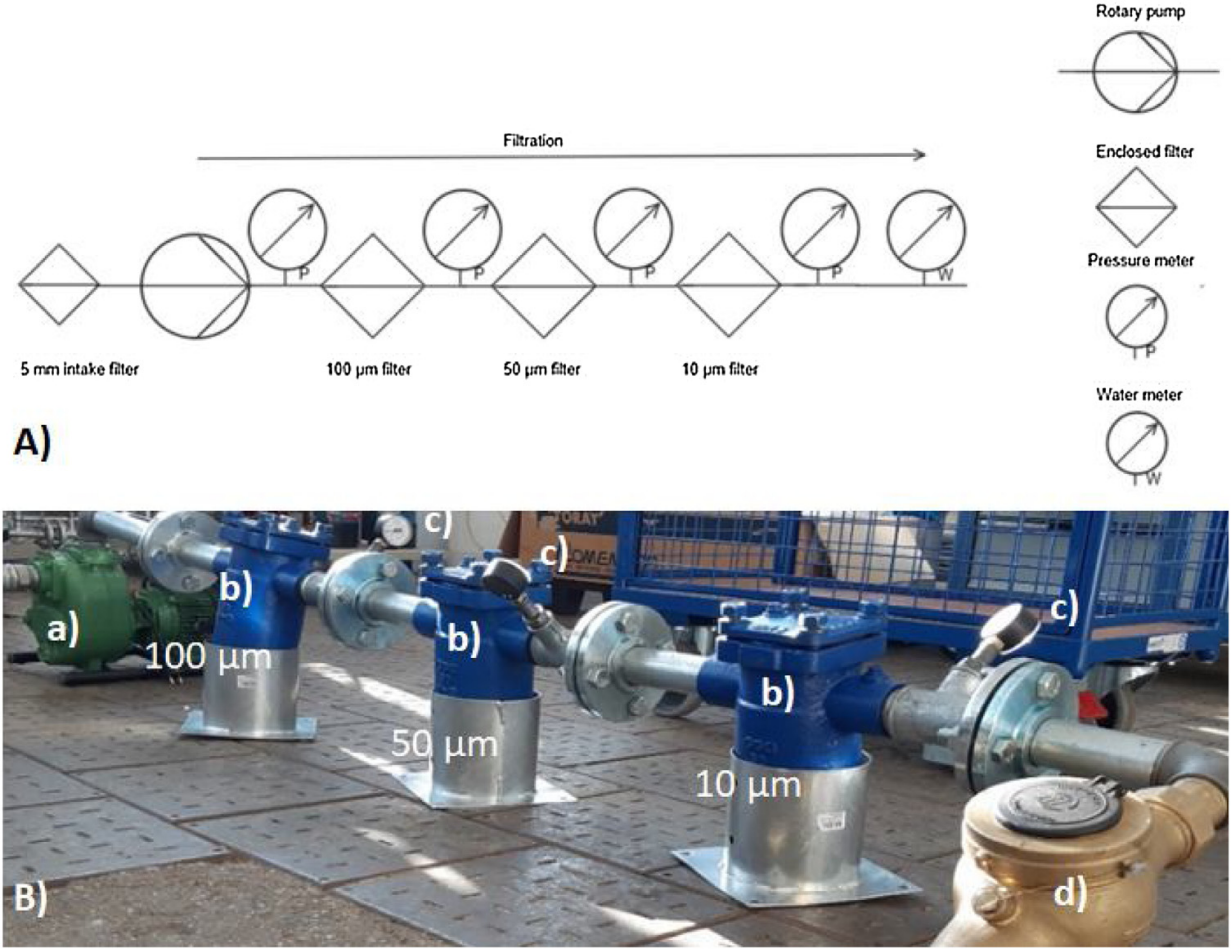
A) Instrumentation diagram for the microplastic filtration plant. B) Cascade filtration for microplastics with rotary pump (a), filter housings (b), pressure meters (c) and a water meter (d).

A cascadic filtration plant has been developed. The rotary pump (Victor Pumpen GmbH; Munich, Germany) the fittings and filter housings are made of cast iron. Flanges and basket filters are made of steel. A diagram of the filtration plant is shown in Fig. 1. On the intake of the rotary pump an additional 5 mm intake-filter is installed. The rotary pump pushes the water through the filters. The filtration plant consists mainly of 3 steel basket filters which are inlaid with steel. Each filter has a height of 19.5 cm and a diameter of 8 cm with a surface area of 485 cm^2^. A detailed part list can be found in the supporting information (Table SI 1).

The following steps were always carried out preparing the MP filtration plant. The filtration plant was rinsed and backwashed with inserted filters by using 1 m^3^ of tap water. Thereafter the basket filters were carefully cleaned with an iron wire brush with a wooden handle (Hornbach Baumarkt AG; Duisburg, Germany)) and rinsed with tap water. The last step consisted of drying the basket filters with a pressure pistol using nitrogen gas (AIR LIQUIDE Deutschland GmbH; Düsseldorf, Germany). The filters were stored outside the filtration plant, covered with aluminum foil preventing contamination by air. The basket filters were weighted by an FKB 30K5DA scale (KERN & SOHN GmbH; Balingen-Frommern, Germany).

#### Validation experiments

Recovery experiments were performed with PE particles with d_50_-values of 120 μm, 70 μm (both VELOX GmbH; Hamburg, Germany) and 25 μm (Celanese Services Germany GmbH; Sulzbach, Germany) for the 100 μm, 50 μm and 10 μm filters, respectively. 10 g of each PE particle size were suspended in 1 m^3^ of tap water within an IBC container and filtered. In addition, the container was refilled with 1 m^3^ of tap water and filtered. The recovery was gravimetrically determined. The filters were weighted before and after filtration using the FKB 30K5DA scale. The recovered particles were weighted into 20-mL ND20 brown screw top vials (IVA Analysentechnik GmbH & Co. K; Meerbusch; Germany) using a MC1 RC210S scale (Sartorius AG; Göttingen, Germany) scale. Py-GC–MS analyses were performed with the recovered particles. This recovery experiment was repeated 3 times.

Experiments to evaluate the basket filter loading and pressure relationship were conducted similarly as the recovery experiments. The previously mentioned particle sizes were added to the corresponding basket filters. To each basket filter 0 g (blank); 0.1 g; 0.2 g; 0.3 g; 0.5 g; 1 g; 2.5 g and 5 g of PE were added. The particles were added directly into the intake nozzle while only one filter was within the filtration plant. After each addition 200 L were filtered and the filter was not removed, thus accumulation of the particles was allowed.

#### WWTP sampling details

The WWTP treats the wastewater of 92,000 population equivalents. The WWTP´s effluent was sampled and a volume of 3500 L was filtered. During this process 200 L were filtered through the 10 μm filter. Thereafter the filter was taken out of the filtration plant by stopping the filtration process. After securing the filter with aluminum foil the filtration process had been continued.

During the filtration process a field blank was prepared in parallel. Basket filters with the same mesh sizes were exposed to air at the sampling site, rinsed with 2 L of tap water and after the filtration covered in aluminum foil. All basket filters were dried in a drying oven UM 600 (Memmert GmbH + Co. KG; Schwabach; Germany) over night at 60 °C.

Thereafter the basket filters were weighted with the FKB 30K5DA scale. Each filter was placed in an 800 mL beaker and the filter surface was physically cleaned with a micro spatula. If this process did not remove the particles from the inner wall the wire brush was used. Thereafter the particles were transferred with a glass funnel into previously weighted 20 mL ND20 brown screw top vials capped with aluminum foil before closing with the screw top. After the particle extraction the empty basket filters and screw top vials were weighted.

### Pyrolysis-GC–MS method

The Py-GC–MS system consisted of a Pyrola 2000 (Pyrol AB; Lund, Sweden) which was provided by Axel Semrau GmbH & Co.KG (Sprockhövel, Germany) and a Thermo Fisher Scientific ISQ GC–MS system (Thermo Fisher Scientific Inc.; Waltham, USA). An OPTIMA 1 MS column (MACHEREY-NAGEL GmbH & CO. KG; Düren, Germany) was installed with ID of 0.25 mm, film thickness of 0.25 μm and length of 30 m. The pyrolysis was online coupled with the split/splitless (SSL) injector of the GC–MS system.

The pyrolysis is equipped with a screw head, holding a platinum filament for sample application. The platinum filament had dimensions of 20 mm × 5 mm. The filament was inserted into the heatable pyrolysis chamber, with a withdrawable glass cell. The platinum filament can reach reliably pyrolysis temperatures (temp.) between 550 °C and 1300 °C within 8 ms.

For all Py-GC–MS measurements the pyrolysis temperature was set to 600 °C with a pyrolysis holding time of 2 s and a chamber temperature of 200 °C. The SSL-injector temp was operated at 280 °C with 1:10 split with a split flow of 10 mL min^−1^ and a column flow of 1 mL min^−1^ of He with 99.999 mol% purity (AIR LIQUIDE Deutschland GmbH; Düsseldorf, Germany). The GC temperature program consisted of a start temp. of 40 °C with a hold time of 4 min, a first temp. ramp with 5 °C min^−1^ till 200 °C and second temperature ramp with 10 °C min^−1^ till 300 °C. The detector transfer line was set to 280 °C and the electron ionization source was set to 250 °C with an ionization energy of 70 eV. The measurements were carried out in full scan mode with a mass range of *m*/*z* 40–450 and a scan time of 0.204 s.

Solid samples were applied to the filament with two micro-spatula and fixated with 5 μL of pure ethanol. The dispersed samples, on the other hand, were applied with an Exmire 10 μL syringe with a blunt tip (ITO CORPORATION; Shizuoka, Japan).

Before pyrolysis was executed of a sample, the pyrolysis chamber was flushed for 10 s with He in order to prevent oxidative conditions during the pyrolysis. The analysis time was one hour.

Calibration samples with a mixture of PS (BS-Partikel GmbH; Mainz, Deutschland) with a d_50_-value of 504 nm and PE (d_50_ of 25 μm) were prepared. The concentrations are shown in SI. Table 4. The respective polymer weights were dispersed in 10 mL ethanol. An ultrasonic finger BANDELIN SONOPLUS HD 2000 (BANDELIN electronic GmbH & Co. KG; Berlin, Germany) was used for 15 min to disperse the polymer particle in ethanol. 2 μL of the internal standard styrene-d_8_ with 98 % purity (Sigma-Aldrich Chemie GmbH; Taufkirchen, Germany) were added to each dispersion. 5 μL of each calibration dispersion were directly applied to the pyrolysis filament. The results of calibration no.1 (s. Table SI 1) were used to calculate the limit of detection (LOD) and limit of quantification (LOQ) using the signal to noise ratio (S/N) of 3 and 10, respectively. The repeatability of the method was tested by measuring the calibration no. 3 three times over the course of three days.

The extracted WWTP samples were ground with a mortar and pestle for homogenization and an aliquot (s. Table 3) was taken with tweezers and a spatula. WWTP samples were weighted in a 70 μL ceramic crucible (Mettler Toledo; Gießen, Germany) with a ME5-F filter-scale (Sartorius AG; Göttingen, Germany). 5 μL of ethanol with the same concentration of internal standard as previously described, were added to the filament.

Blank measurements were conducted. Lab air blanks were analyzed by opening and closing the pyrolysis chamber. Furthermore, the used ethanol was analyzed. To avoid carry over the pyrolysis filament and glass cell were cleansed by a blowtorch after each measurement. This procedure was conducted with the capillary inserted to the SSL-injector with helium flowing through preventing the capillary from melting.

For analysis Thermo Excalibur 2.1 0.114 acquisition software was used. The raw data file was converted by the inbuilt file converter into a cdf. file. Thereafter the cdf. file was converted into a d. file and evaluated with Agilent´s Masshunter 10 qualitative analysis software Version 10.0.10305.0, which allows data evaluation on Windows 10 systems.

### Filtration plant validation results

The combined recovery rates for loss of particles within the filtration plant itself and the particle extraction from the basket filters yielded the following results. In the 100 μm filter a recovery of 87 % ± 2 % was observed. In the 50 μm and 10 μm filters recovery rates of 85 % ± 2 % and 88 % ± 2 %, respectively, were found. Bannick et al. [1] found a recovery of 81 % for PE, therefore the achieved results are within reason.

**Fig. 2:**
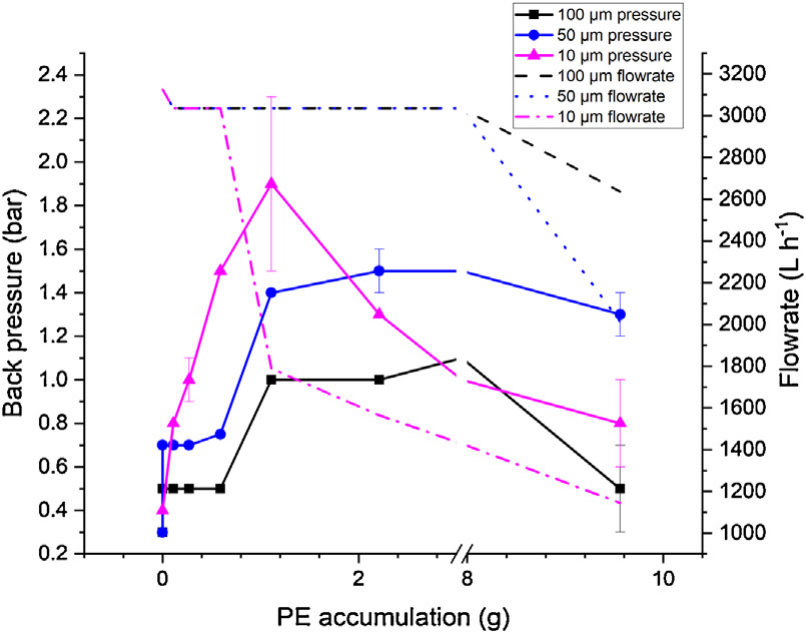
Back pressure (bar) and flowrate (L h^−1^) in dependence of particle accumulation within the 100 μm, 50 μm and 10 μm filters. To each filter stepwise 0 g (blank); 0.1 g; 0.2 g; 0.3 g; 0.5 g; 1 g; 2.5 g and 5 g PE with the respective particle sizes of 120 μm, 70 μm and 25 μm were added directly into the intake nozzle. Between each particle addition 200 L of water were filtered from an IBC-container. During this filtration the back pressure was recorded of the single respective filter within the filtration plant.

During these experiments a MP water suspension was filtered and as more water was filtered an increase of the back pressure was observed. An unwanted effect during the MP filtration is cake-filtration. Therefore, the influence between particle loading and back pressure was investigated. The results are shown in Fig. 2. The filters exhibit a base resistance to the filtration medium of water [2]. This results in a base back pressure of 0.3 bar for the 100 μm and 50 μm filters and 0.4 bar for the 10 μm filter. With addition of particles to the filters the back pressure increases as the particles block the mesh of the basket filters and thus increase the resistance to the water flow. Until the addition of 0.6 g of PE to the 100 μm and 50 μm filters, the back pressure for both filters is stable at 0.5 bar and 0.7 bar, respectively. After further adding of 0.5 g PE the back pressure increases to 1 bar for the 100 μm filter and to 1.4 bar for the 50 μm filter. This might be caused by a filter cake build up as the filter cake itself exhibits a filtration resistance in addition to particle blocking of the filter mesh [3]. The back pressure of the 10 μm filter increases significantly after the addition of 0.1 g of PE from 0.4 bar to 0.8 bar. After adding a total amount of 1 g of PE the pressure rose up to 1.8 bar. At this point a vial was sucked into the filtration plant, which probably caused the pressure spike of 1.8 bar, which explains why the flowrate was still constant. For this filter no constant back pressure can be seen for all chosen particle loadings.

With a further increase of the particle loading the back pressure rises for all three filters above 1.5 bar and the flow rate (L h^−1^) decreases significantly thereafter. The output of the pump allows a maximum back pressure of 1.5 bar. As this value is exceeded for all filters less water is filtered as pump performance decreases. The anomaly observed when the 10 μm filter was tested originated from the sucked in vial. To reduce cake filtration, the filtration is stopped when pressures of 0.5 bar, 0.7 bar and 0.8 bar for the 100 μm, 50 μm and 10 μm filters are exceeded, respectively.

### Pyrolysis-GC–MS results

For identifying PS and PE the characteristic pyrolysis decomposition products were identified and are shown with retention time and characteristic fragment ions in Table 1. For PS styrene (PS1), 2,4-Diphenyl-1-butene (PS2) and 2,4,6-Triphenyl-1-hexene (PS3) were identified as characteristic pyrolysis products. If PS1 and PS2 are detected in a sample PS was identified and PS3 is used for quantification. PS1 is a compound which could originate from other sources than microplastic and therefore is not a reliable identifier for PS. For PE 1,12-Tridecadiene (PE1), 1,13-Tetradecadiene (PE2) and 1,14-Pentadecadiene (PE3) were identified. PE3 is used for quantification of PE, if PE1, PE2 and PE3 were detected. The found compounds for PS and PE are reported in literature. Fischer et al. (2017) used a curie-point Py-GC–MS for pyrolysis and reported the same decomposition compounds for PS [4]. Dümichen et al. (2015, 2017, 2019) reported the same compounds with the same characteristic fragment masses using a TED-GC–MS [[5], [6], [7]]. Fries et al. (2013) used a Py-GC–MS and identified PS1 and PE1 [8].

**Table 1:** Selected pyrolysis products for PS and PE identification. Characteristic fragment ions *m*/*z* and retention times (min) for each pyrolysis product are presented with characteristic fragment ions and the respective intensity ratios.

Polymer	Pyrolysis product name		Characteristic fragment ions *m/z (i.r.)*	Retention time (min)
PS	PS1	Styrene	51 (12 %); 78 (30 %); 104 (100 %)	8.49
	PS2	2,4-Diphenyl-1-butene	91 (100 %); 104 (16 %); 130 (11 %); 208 (13 %)	31.64
	PS3	2,4,6-Triphenyl-1-hexene	91 (100 %); 117 (30 %); 194 (15 %); 207 (23 %)	42.42
PE	PE1	1,12-Tridecadiene	55 (100 %); 81 (74 %); 67 (62 %); 95 (40 %)	21.52
	PE2	1,13-Tetradecadiene	81 (74 %); 95 (40 %); 109 (20 %)	24.22
	PE3	1,14-Pentadecadiene	55 (100 %); 81 (60 %); 95 (38 %); 109 (15 %)	26.75

*i.r. intensity ratio compared to the largest peak in the fragment ion pattern.

Preceding the analysis of the WWTP samples a calibration for PS and PE was conducted. The results are shown in Table 2. The R^2^ for all compounds is above 0.9 except for PS2 with a R^2^ 0.8358. The relative standard deviations for all compounds equal or below 11 %. The limit of detection (LOD) and limit of quantification (LOQ) are comparable to values reported in literature. Dümichen et al. [7] analyzed solid particles and weighted these into the TED-GC–MS. The reported LOQs for PS1, PS2, PS3, PE1, PE2 and PE3 were: 0.135 μg, 0.195 μg, 0.23 μg, 11.4 μg, 9.55 μg and 12.2 μg, respectively. Fischer et al. [9] dissolved PS and found LOD/LOQ for PS1 with 0.003 μg/0.016 μg and for PS3 0.059 μg/0.282 μg.

**Table 2:** Linear regression correlation coefficient (Corr. R^2^), limit of detection (LOD) and limit of quantification (LOQ) of the characteristic pyrolysis products of PS and PE are shown. For all analyses the pyrolysis took place at 600 °C for 2 s with a pyrolysis chamber temperature of 200 °C. The injector temperature was set to 250 °C with a split flow of 10 mL min^−1^ and a 1:10 split.

Char. pyr. prod. PS	Corr. R^2^	LOD (μg)	LOQ (μg)	Rel. std. dev.	Char. pyr. prod. PE	Corr. R^2^	LOD (μg)	LOQ (μg)	Rel. std dev.
Styrene	0.9606	0.0004	0.001	3	1,12-Tridecadiene	0.9988	0.4	1.2	9
2,4-Diphenyl-1-butene	0.8358	0.009	0.03	10	1,13-Tetradecadiene	0.9998	0.3	1.0	11
2,4,6-Triphenyl-1-hexene	0.9968	0.009	0.03	9	1,14-Pentadecadiene	0.9868	0.3	1.1	10

After weighting the WWTP residues extracted from the filters aliquots of the samples were taken and analyzed. The samples were not pretreated besides homogenization. Table 3 shows the results of the sampling and analysis. Most particles were extracted from the 100 μm filter with 0.170 g. As the mesh size decreases in the 50 μm and 10 μm filters the found particle mass decreased with 0.060 g and 0.008 g, respectively. As the particle size decreases the overall mass of the particles decreases. A comprehensive particle size distribution cannot be obtained with the Py-GC–MS, only spectroscopic methods like RAMAN and FT-IR are suitable for this propose. However, the presented Py-GC–MS method is best suited for the determination of mass and entry balances. For analysis from the 100 μm, 50 μm and 10 μm filter 0.2 %, 0.6 % and 4.75 % aliquots were taken, respectively. Within the analyzed aliquots from the filters only PS was detected. In the samples PE was not found, since the LODs for PE1, PE2 and PE3 are significantly higher compared to the characteristic pyrolysis products of PS. The aliquot sample from the 100 μm filter contained 0.43 μg PS. The calculated percentage of PS in the aliquot sample was therefore 0.12 % (wt%), the remainder were organic- and inorganic compounds. In the 50 μm and 10 μm filters PS percentages of 0.05 % and 0.007 % were observed, respectively. The combined effluent concentration of PS from the WWTP is 0.072 mg m^−3^.

**Table 3:** Py-GC–MS analysis results of the effluent sampling of the WWTP. Aliquots of the recovered samples from the 100 μm, 50 μm and 10 μm filter were transferred to the platinum filament and fixated with 5 μL of ethanol, containing 1 μg absolute styrene-d_8_. The samples were pyrolyzed at 600 °C for 2 s with a split flow of 10 mL min^−1^ and a 1:10 split. The pyrolysis chamber temperature was set to 200 °C and the injector temperature to 250 °C. PS3 (2,4,6-Triphenyl-1-hexene) was used for identification of PS.

Filter mesh size	Found particle mass in corr. filter (mg)	Aliquot mass from filter (mg)	Identified polymer	Mass PS found in aliquot (mg)	Mass PS per m^3^ of WWTP effluent (mg)
100 μm	170	0.35	PS	4.30E-04	0.060
50 μm	60	0.37	PS	2.00E-04	0.009
10 μm	8	0.38	PS	3.00E-05	0.003

*A volume of 3500 L was filtered through the 100 μm and 50 μm filters. 200 L were filtered through the 10 μm filter.

## Conclusion

In this work a fast and reliable quantitative method for sampling and analytical determination of microplastic in surface and waste water samples is presented. For this purpose, a cast iron based cascadic MP filtration plant using steel filters with mesh sizes of 100 μm, 50 μm and 10 μm has been developed, validated and tested in the field.

For the MP analysis a Py-GC–MS based method was developed. Blank measurements of the lab air were negative and with the presented clean up steps no carry overs were detected. A novel approach for calibration was chosen in the context of MP analysis using mixed PS and PE dispersions and styrene d8 as internal standard. With a relative standard deviation of around 11 % the calibrations using PS and PE dispersed in ethanol allow a fast simple and viable sample application onto the small platinum filament. LOQ´s and LOD´s of PS and PE exceed the values achieved by a TED-GC–MS, although these values were achieved without calibrating in matrix. The maximum sample amount introduced into the Py-GC–MS is around 300–400 μg of sample. The combination of both methods allowed sampling and analysis of a WWTP effluent without sample preparation besides the sample extraction and drying overnight. Therefore, the presented method combination allows a fast analysis of microplastics from water bodies.
