# Supplementary material for: Identification of microplastics in wastewater after cascade filtration using Pyrolysis-GC–MS
Source: MethodsX. 2019 Dec 19;7:100778. doi: 10.1016/j.mex.2019.100778 (PMC7152672; doi:10.1016/j.mex.2019.100778)
Supplement: Supplementary file 1 [file mmc1.pdf]

Supplementary Information

1. Supplementary material

Table SI 1: Part list with supplier information for the cascadic MP filtration plant.

Part Name	Supplier information
SG 40 Rotary pump DN 40	Victor Pumpen GmbH (Kirchheim, Germany)
KMF filter housing DN 40	Krone Filter Solutions GmbH (Oyten, Germany)
KMF basket filter 100 µm; 50 µm;10 µm with height 19.5 cm, diameter 8 cm	Krone Filter Solutions GmbH (Oyten, Germany)
5 mm intake filter DN 25	Hornbach Baumarkt AG (Duisburg, Germany)
Pressure sensor DN 8	Stabilo Sanitärgroßhandel GmbH (Bad Windsheim, Germany)
T interface DN 40 to DN 20	TE Fittings GmbH (Hattingen, Germany)
interface screw pipe DN 20 to DN 8	TE Fittings GmbH (Hattingen, Germany)
Fittings DN 8	TE Fittings GmbH (Hattingen, Germany)
Flange DN 40	Stabilo Sanitärgroßhandel GmbH (Bad Windsheim, Germany)
Water meter DN 40	Stabilo Sanitärgroßhandel GmbH (Bad Windsheim, Germany)
Fittings DN 40	TE Fittings GmbH (Hattingen, Germany)
Screw pipe DN 40	TE Fittings GmbH (Hattingen, Germany)
Panzer pipe DN 40	Stabilo Sanitärgroßhandel GmbH (Bad Windsheim, Germany)
Panzer pipe DN 25	Stabilo Sanitärgroßhandel GmbH (Bad Windsheim, Germany)
Interface screw pipe DN 40 to DN 25	TE Fittings GmbH (Hattingen, Germany)
Hemp	TE Fittings GmbH (Hattingen, Germany)
Sealing paste	TE Fittings GmbH (Hattingen, Germany)

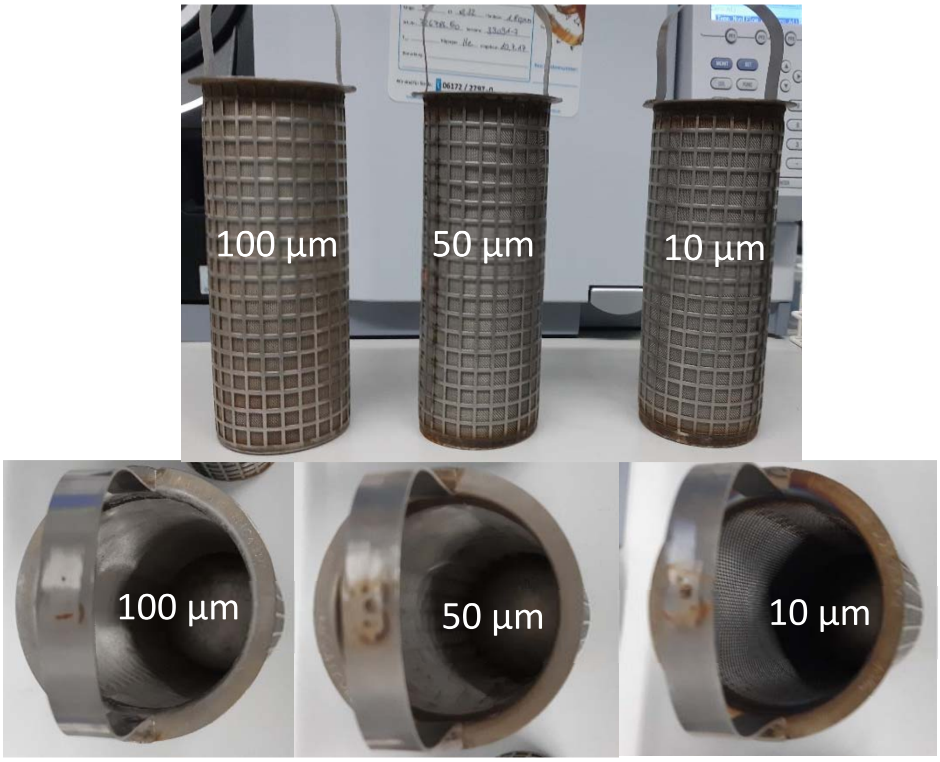

Figure SI 1: Outside and inside depiction of the KMF basket filters with mesh sizes of 100 µm, 50 µm and 10 µm. Each filter has a height of 19.5 cm with a diameter of 8 cm. The filtration surface is 485 cm^2.

Table SI 2: Calibration concentrations of the PS d_{50} 504 nm and PE d_{50} 25 µm jointed 10 mL ethanol dispersion used for the Py-GC-MS. 5 µL of the dispersion were applied to the platinum filament. Additionally, the 5 µL dispersion contained 1 µg absolute of the internal standard styrene d_8. The calibration samples were pyrolyzed at 600 °C for 2 s and a pyrolysis chamber temperature of 200 °C

Calibration No.	Absolute mass applied (PS)	Absolute mass applied (PE)
**1**	0.1 µg	1 µg
**2**	0.5 µg	2 µg
**3**	1 µg	3 µg
**4**	1.5 µg	4 µg

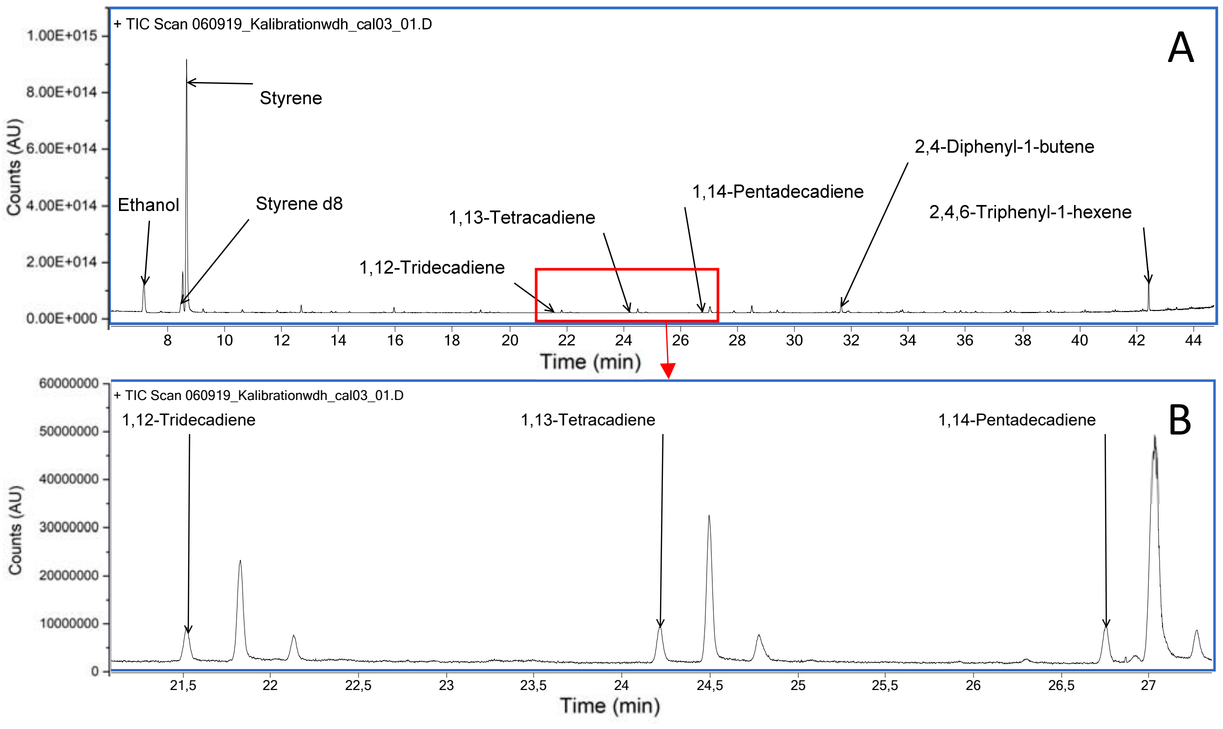

Figure SI 2: Total Ion Chromatogram (TIC) of calibration No. 3 of the PS d_{50} 504 nm and PE d_{50} 25 µm jointed 10 mL ethanol dispersion used for the Py-GC-MS. 5 µL of the dispersion were applied to the platinum filament. Additionally, the 5 µL dispersion contained 1 µg absolute of the internal standard styrene d_8. The calibration samples were pyrolyzed at 600 °C for 2 s and a pyrolysis chamber temperature of 200 °C. A) Characteristic pyrolysis compounds are presented for PS (Styrene, 2,4-Diphenyl-1-butene, 2,4,6-Triphenyl-1-hexene) and PE (1,12-Tridecadiene, 1,13-Tetradecadiene, 1,14-Pentadecadiene). B) Zoom of Total Ion Chromatogram (TIC) of calibration No. 3 from part A, with a focus on PE pyrolysis products. The PS d_{50} 504 nm and PE d_{50} 25 µm jointed 10 mL ethanol dispersion had been used for the Py-GC-MS. 5 µL of the dispersion were applied to the platinum filament. Additionally, the 5 µL dispersion contained 1 µg absolute of the internal standard styrene d_8. The calibration samples were pyrolyzed at 600 °C for 2 s and a pyrolysis chamber temperature of 200 °C.

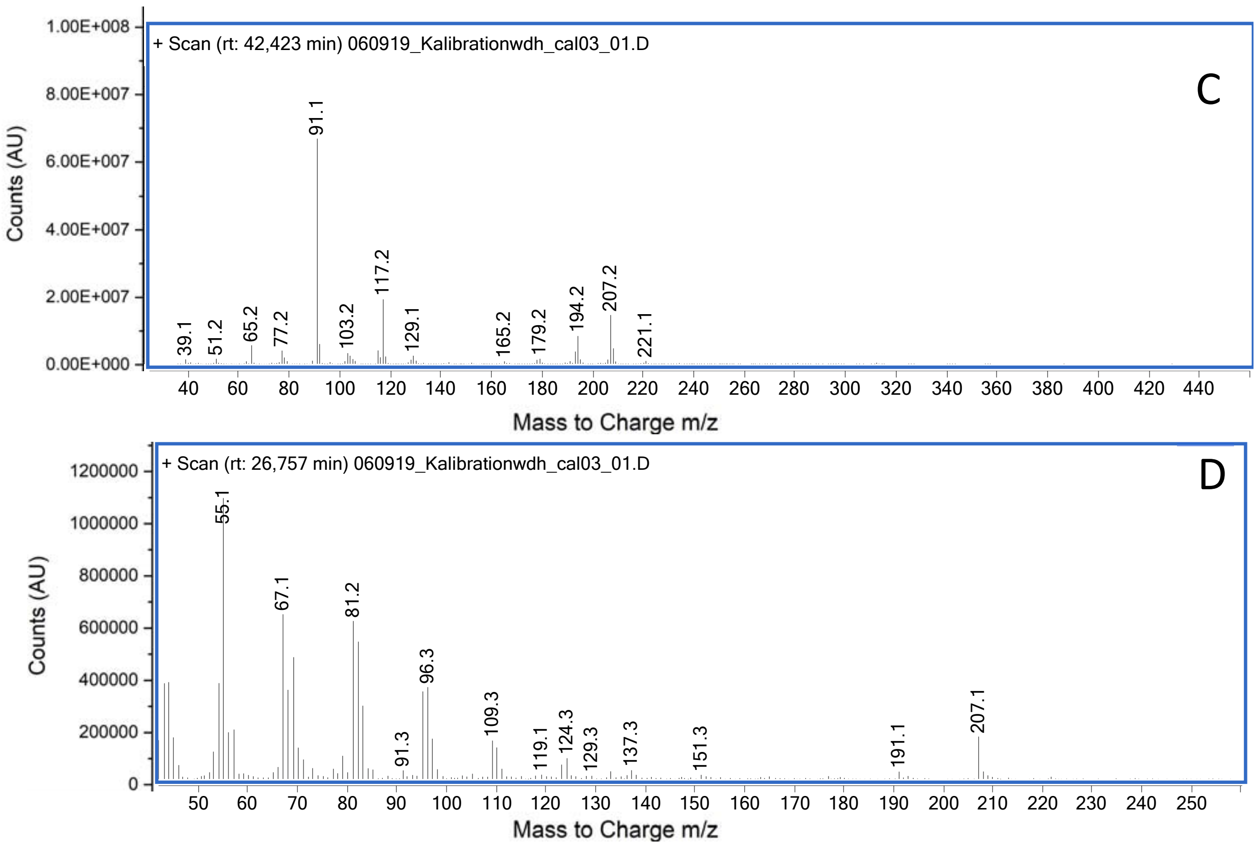

Figure SI 3: C) Obtained mass spectrum of 2,4,6-Triphenyl-1-hexene from Figure SI 2 (calibration No. 3) with characteristic masses of 91 (100%), 117 (30%), 194 (15%) and 207 (23%).D) Obtained mass spectrum of from Figure SI 2 (calibration No. 3) 1,14-Pentadecadiene with characteristic masses of 55 (100%), 81 (60%), 95 (38%) and 109 (15%).

2. Background

The first description of small polystyrene spheres in the ocean took place in 1972 by Carpenter et. al. [1]. Since these investigations, the topic of microplastic (MP) has gained in importance [2–9]. MP detected in the aquatic environment are either primary or secondary in nature [10]. Primary MPs are intentionally produced, while secondary MPs are formed by degradation from macroplastics [11]. Sampling methods for MP depends on the investigated medium and the detection method [12]. Many sampling procedures for MP focus on the marine and fresh water environment [13,14]. In these studies manta trawl (300 µm-333 µm), plankton (80 µm-333 µm)- and neuston nets (333 µm mesh size) with a variety of mesh sizes are reported [6,14–17]. Several studies also employed pumping systems with filters to sample MP from water bodies using stainless steel sieves (250 µm; 10 µm mesh size) [18,19].

For WWTPs similar techniques have been used as in the marine environments. Additionally, two studies presented in 2016 a cascadic microplastic filtration for waste water (WW), using pan (400 µm mesh size)- and tyler (20 µm mesh size) sieve filters, respectively [20,21]. A common problem of all used filtration methods is cake filtration. This occurs during filtration when the filter mesh is clogged due to high particle load [13] and alters the volumetric flow rate of the water [22]. In addition, smaller particles accumulate on this filter cake, making a representative and quantitative sampling of the targeted particle size fraction difficult. In order to address the cake filtration Bannick et al.(2019) validated a similar pan sieve approach as presented by Carr et al.(2016) and Sutton et al. (2016) [13,20,21]. The study of Bannick et al. (2019) focused on challenging aspects of the comparability of mesh sizes between studies, the filtration of large volumes and the classes of sampling sizes for thermo-analytical detection methods [13].

The latter aspect addresses the MP size information, which cannot be measured by thermo-analytical detection methods. The predominant thermo-analytical detection methods for MP is the novel thermogravimetric (TGA) and thermal desorption (TD) based thermal extraction desorption gaschromatography mass spectrometry (TED-GC-MS) [23–27] and the Py-GC-MS-systems with various pyrolizers [28–33]. Both instrument types differ in their analytical workflow and sample application. The TED-GC-MS is a sorption- and desorption based method [24]. Thus, samples are indirectly introduced to the GC-MS-system via an adsorbent that is loaded with the pyrolysis products [24].

Py-GC-MS-systems directly introduce pyrolysis gases into the GC-MS-system. This restricts the maximum amount, which can be introduced into the system without overloading the GC-column.

Hidalgo et al. (2012) stated that the MP sampling method needs to be designed with the analysis method in mind [12]. Therefore,

the aim of this study is to present a cascadic filtration-based sampling method, allowing for a particle size fractionation. Thus, a quantitative sampling with correct size information from each fraction is possible. Each size fraction is analyzed with a platinum filament-based pyrolysis-GC/MS, quantifying the MP mass. The combined sampling and analysis method allows a mass determination and a size classification for MP within the sample.

3. Additional information

In literature the focus is on either of the methods regarding MP. The sampling method aims to allow a fast cost effective and simple MP filtration with a single pump set-up. Furthermore, the filtration plant set-up is according to the propositions made by Bannick et al. (2019) regarding the cascadic character and mesh sizes [1]. Compared to sieve filtrations [1,10,11] and net filtration [12–16] the basket filters and subsequently MP samples are enclosed, thus contaminations via air do not occur during filtration. Therefore, MP samples are suited for analysis via micro

spectroscopic- and thermo-analytical methods. In the currently presented form the filtration plant needs to be assembled on site, requiring leak checks.

The used pyrolysis unit can be coupled to an existing GC-MS-system with little modifications and represents a simple alternative to TED-GC-MS. The combination of LOQ´s and LOD´s of PS and PE exceed the values achieved by a TED-GC-MS, although these values were achieved without calibrating in matrix. The maximum sample amount introduced into the Py-GC-MS is around 300 to 400 µg of sample, whereas larger sample quantities can be applied onto the TED-GC-MS. The combination of both methods allowed sampling and analysis of a WWTP effluent without sample preparation besides the sample extraction and drying overnight. Therefore, the presented method combination allows a fast analysis of microplastics from water bodies.

4. References

[1] Carpenter, E. J., Anderson, S. J., Harvey, G. R., Miklas, H. P., Peck, B. B. (1972). Polystyrene Spherules in Coastal Waters. Science **178**/4062, 749–750.

[2] Moore, C. J. (2008). Synthetic polymers in the marine environment: A rapidly increasing, long-term threat. Environmental research **108**/2, 131–139.

[3] Barnes, D. K. A., Galgani, F., Thompson, R. C., Barlaz, M. (2009). Accumulation and fragmentation of plastic debris in global environments. Philosophical transactions of the Royal Society of London. Series B, Biological sciences **364**/1526, 1985–1998.

[4] Thompson, R. C., Swan, S. H., Moore, C. J., Vom Saal, F. S. (2009). Our plastic age. Philosophical transactions of the Royal Society of London. Series B, Biological sciences **364**/1526, 1973–1976.

[5] Ryan, P. G., Moore, C. J., van Franeker, J. A., Moloney, C. L. (2009). Monitoring the abundance of plastic debris in the marine environment. Philosophical transactions of the Royal Society of London. Series B, Biological sciences **364**/1526, 1999–2012.

[6] Andrady, A. L. (2011). Microplastics in the marine environment. Marine pollution bulletin **62**/8, 1596–1605.

[7] Koelmans, A. A., Mohamed Nor, N. H., Hermsen, E., Kooi, M., Mintenig, S. M., France, J. de (2019). Microplastics in freshwaters and drinking water: Critical review and assessment of data quality. Water research **155**, 410–422.

[8] Triebskorn, R., Braunbeck, T., Grummt, T., Hanslik, L., Huppertsberg, S., Jekel, M., Knepper, T. P., Krais, S., Müller, Y. K., Pittroff, M., Ruhl, A. S., Schmieg, H., Schür, C., Strobel, C., Wagner, M., Zumbülte, N., Köhler, H.-R. (2018). Relevance of nano- and microplastics for freshwater ecosystems: a critical review. TrAC Trends in Analytical Chemistry.

[9] Renner, G., Schmidt, T. C., Schram, J. (2018). Analytical methodologies for monitoring micro(nano)plastics. Which are fit for purpose? Current Opinion in Environmental Science & Health **1**, 55–61.

[10] Alimi, O. S., Farner B., J., H., L. M., Tufenkji, N. (2018). Microplastics and Nanoplastics in Aquatic Environments. Aggregation, Deposition, and Enhanced Contaminant Transport. Environmental science & technology 52, 1704-1724.

[11] Thompson, R. C., Olsen, Y., Mitchell, R. P., Davis, A., Rowland, S. J., John, A. W. G., McGonigle, D., Russell, A. E. (2004). Lost at sea. Where is all the plastic? Science **304**/5672, 838.

[12] Hidalgo-Ruz, V., Gutow, L., Thompson, R. C., Thiel, M. (2012). Microplastics in the marine environment. A review of the methods used for identification and quantification. Environmental science & technology **46**/6, 3060–3075.

[13] Bannick, C. G., Szewzyk, R., Ricking, M., Schniegler, S., Obermaier, N., Barthel, A. K., Altmann, K., Eisentraut, P., Braun, U. (2019). Development and testing of a fractionated filtration for sampling of microplastics in water. Water research **149**, 650–658.

[14] Li, J., Liu, H., Paul Chen, J. (2018). Microplastics in freshwater systems. A review on occurrence, environmental effects, and methods for microplastics detection. Water research **137**, 362–374.

[15] Doyle, M. J., Watson, W., Bowlin, N. M., Sheavly, S. B. (2011). Plastic particles in coastal pelagic ecosystems of the Northeast Pacific ocean. Marine environmental research **71**/1, 41–52.

[16] Goldstein, M. C., Titmus, A. J., Ford, M. (2013). Scales of Spatial Heterogeneity of Plastic Marine Debris in the Northeast Pacific Ocean. PLoS ONE **8**/11, e80020.

[17] Ter Halle, A., Jeanneau, L., Martignac, M., Jardé, E., Pedrono, B., Brach, L., Gigault, J. (2017). Nanoplastic in the North Atlantic Subtropical Gyre. Environmental science & technology **51**/23, 13689–13697.

[18] Lusher, A. L., Burke, A., O'Connor, I., Officer, R. (2014). Microplastic pollution in the Northeast Atlantic Ocean: Validated and opportunistic sampling. Marine pollution bulletin **88**/1-2, 325–333.

[19] Enders, K., Lenz, R., Stedmon, C. A., Nielsen, T. G. (2015). Abundance, size and polymer composition of marine microplastics ≥10μm in the Atlantic Ocean and their modelled vertical distribution. Marine pollution bulletin **100**/1, 70–81.

[20] Carr, S. A., Liu, J., Tesoro, A. G. (2016). Transport and fate of microplastic particles in wastewater treatment plants. Water research **91**, 174–182.

[21] Sutton, R., Mason, S. A., Stanek, S. K., Willis-Norton, E., Wren, I. F., Box, C. (2016). Microplastic contamination in the San Francisco Bay, California, USA. Marine pollution bulletin **109**/1, 230–235.

[22] Evans, M. S., Sell, D. W. (1985). Mesh size and collection characteristics of 50-cm diameter conical plankton nets. Hydrobiologia **122**/2, 97–104.

[23] Dümichen, E., Barthel, A.-K., Braun, U., Bannick, C. G., Brand, K., Jekel, M., Senz, R. (2015). Analysis of polyethylene microplastics in environmental samples, using a thermal decomposition method. Water research **85**, 451–457.

[24] Dümichen, E., Eisentraut, P., Celina, M., Braun, U. (2019). Automated thermal extraction-desorption gas chromatography mass spectrometry: A multifunctional tool for comprehensive characterization of polymers and their degradation products. Journal of chromatography. A **1592**, 133–142.

[25] Dümichen, E., Braun, U., Senz, R., Fabian, G., Sturm, H. (2014). Assessment of a new method for the analysis of decomposition gases of polymers by a combining thermogravimetric solid-phase extraction and thermal desorption gas chromatography mass spectrometry. Journal of chromatography. A **1354**, 117–128.

[26] Dümichen, E., Eisentraut, P., Bannick, C. G., Barthel, A.-K., Senz, R., Braun, U. (2017). Fast identification of microplastics in complex environmental samples by a thermal degradation method. Chemosphere **174**, 572–584.

[27] Eisentraut, P., Dümichen, E., Ruhl, A. S., Jekel, M., Albrecht, M., Gehde, M., Braun, U. (2018). Two Birds with One Stone—Fast and Simultaneous Analysis of Microplastics: Microparticles Derived from Thermoplastics and Tire Wear. Environ. Sci. Technol. Lett. **5**/10, 608–613.

[28] Fries, E., Dekiff, J. H., Willmeyer, J., Nuelle, M.-T., Ebert, M., Remy, D. (2013). Identification of polymer types and additives in marine microplastic particles using pyrolysis-GC/MS and scanning electron microscopy. Environmental science. Processes & impacts **15**/10, 1949–1956.

[29] Fischer, M., Scholz-Böttcher, B. M. (2019). Microplastics analysis in environmental samples – recent pyrolysis-gas chromatography-mass spectrometry method improvements to increase the reliability of mass-related data. Anal. Methods **11**/18, 2489–2497.

[30] Fischer, M., Scholz-Böttcher, B. M. (2017). Simultaneous trace identification and quantification of common types of microplastics in environmental samples by pyrolysis-gas chromatography-mass spectrometry. Supporting Information. Environmental science & technology **51**/9, 5052–5060.

[31] Wendt-Potthoff, K., Imhof, H., Wagner, M., Primpke, S., Fischer, D., Scholz-Böttcher, B. M., Laforsch, C. (2017). Mikroplastik in Binnengewässern. V. Strukturelle Veränderungen und Belastungen von Gewässern. https://onlinelibrary.wiley.com/doi/full/10.1002/9783527678488.hbal2016003 (letzter Zugriff am 30.5.2018).

[32] Tsuge, S., Ohtani, H., Watanabe, C. (2011). Pyrolysis - GC/MS data book of synthetic polymers. Pyrograms, thermograms and MS of pyrolyzates, 1. Aufl. Elsevier, Amsterdam.

[33] Zhou, X.-X., Hao, L.-T., Wang, H.-Y.-Z., Li, Y.-J., Liu, J.-F. (2019). Cloud-Point Extraction Combined with Thermal Degradation for Nanoplastic Analysis Using Pyrolysis Gas Chromatography-Mass Spectrometry. Analytical chemistry **91**/3, 1785–1790.
